# Supplementary material for: Revealing the Impact of Genomic Alterations on Cancer Cell Signaling with an Interpretable Deep Learning Model
Source: Cancers (Basel). 2023 Jul 29;15(15):3857. doi: 10.3390/cancers15153857 (PMC10416927; doi:10.3390/cancers15153857)
Supplement: Supplementary file 1 [file cancers-15-03857-s001.zip › cancers-2509152-supplementary.pdf]

Supporting information

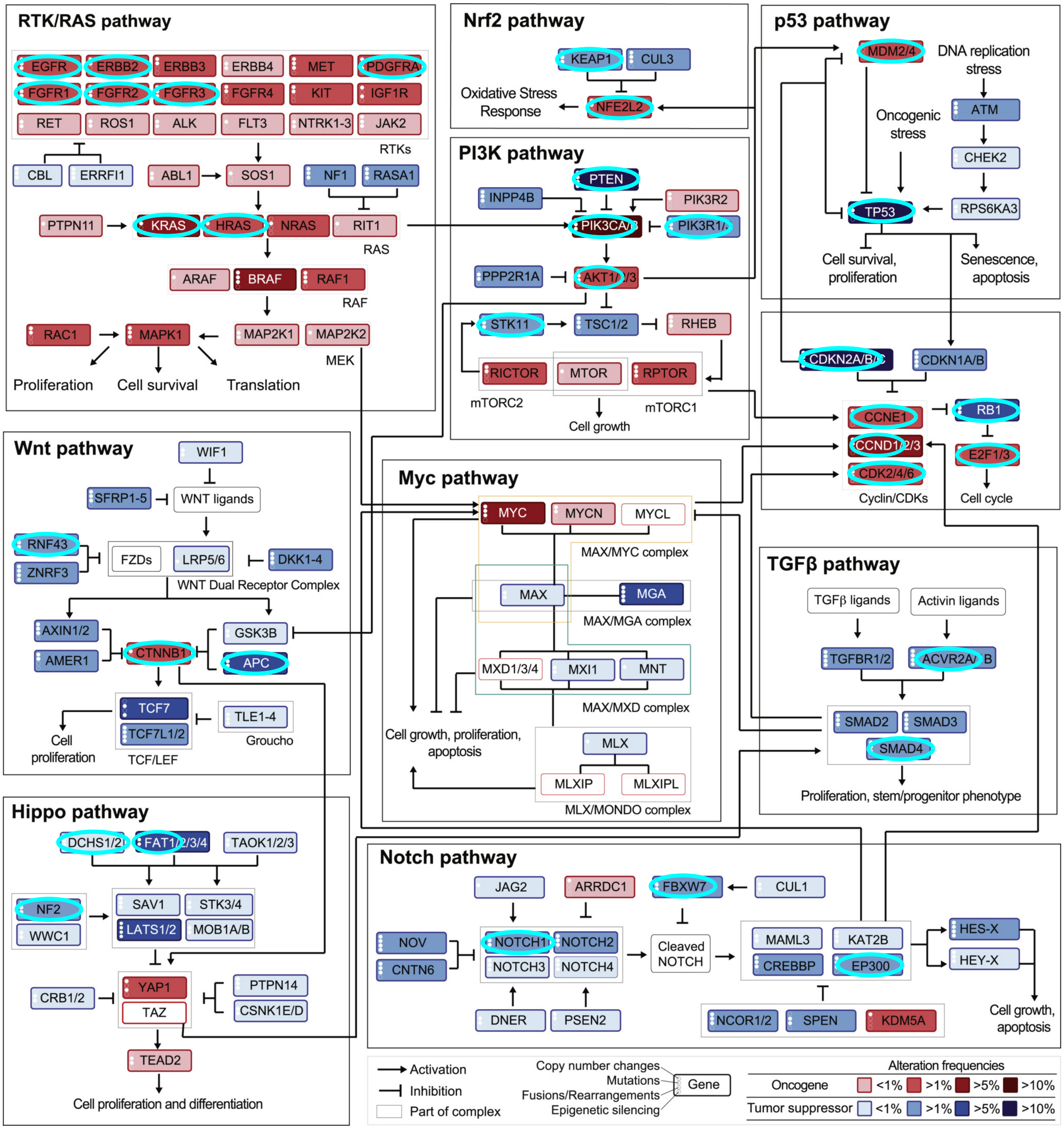

Figure S1: **Genes in SGA Dataset that Overlap with Sanchez-Vega et al., 2018.** This is Figure S1 from Sanchez et al., 2018: "Curated Pathways Including Cross-Cross Pathway Interactions" [?]. Circled in cyan are all genes from these pathways that are in our SGA dataset. We are treating these pathways as ground truth for comparison purposes. The cyan circles have been added to the original figure.
